# Supplementary material for: Food‐Activated Microneedle Sensor for Real‐Time, Colorimetric Spoilage Monitoring of Pre‐Packaged Food
Source: Adv Sci (Weinh). 2025 Nov 12;13(6):e12602. doi: 10.1002/advs.202512602 (PMC12866867; doi:10.1002/advs.202512602)
Supplement: Supplementary file 1 — Supporting Information [file ADVS-13-e12602-s001.docx]

**Supplementary Information**

**Food-activated Microneedle Sensor for Real-time, Colorimetric Spoilage Monitoring of Pre-packaged Food**

*Shadman Khan^†a,b^, Akansha Prasad^†b^, Mahum Javed^†b^, Roderick Maclachlan^c^, Carlos D. M. Filipe*^d^, Tohid F. Didar^*b,e^*

^a^ School of Environmental Sciences, University of Guelph, Guelph, ON N1G 2W1, Canada.

^b^ School of Biomedical Engineering, McMaster University, Hamilton, ON L8S 4L8, Canada.

^c^ Department of Engineering Physics, McMaster University, Hamilton, ON L8S4L7, Canada.

^d^ Department of Chemical Engineering, McMaster University, Hamilton, ON L8S 4L7, Canada.

^e^ Department of Mechanical Engineering, McMaster University, Hamilton, ON L8S 4L7, Canada.

^†^ These authors contributed equally.

^*^To whom correspondence should be addressed. Email: filipec@mcmaster.ca, didart@mcmaster.ca


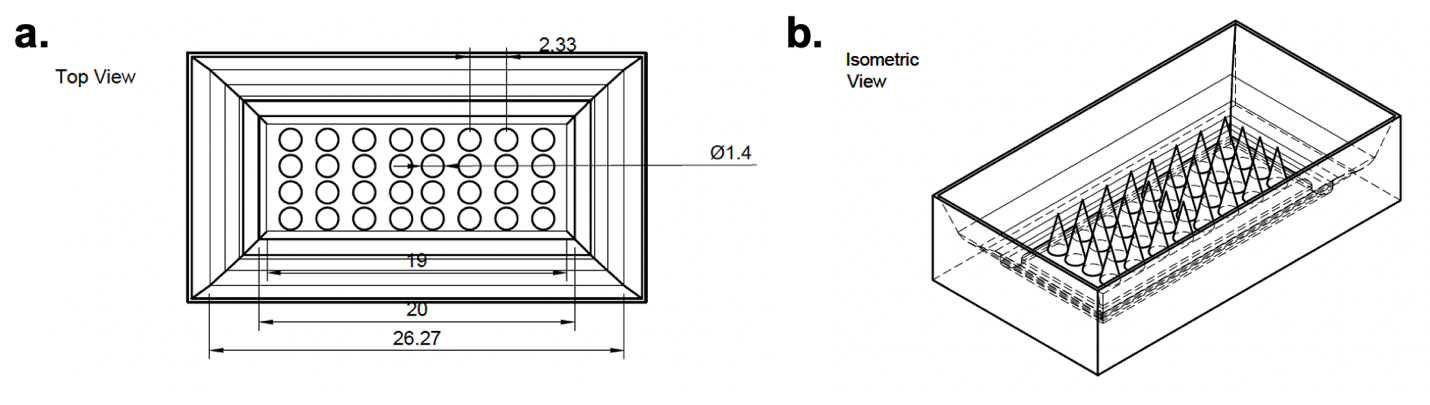


**Figure S1.** Schematic illustrations of master mold fabricated *via* stereolithography.


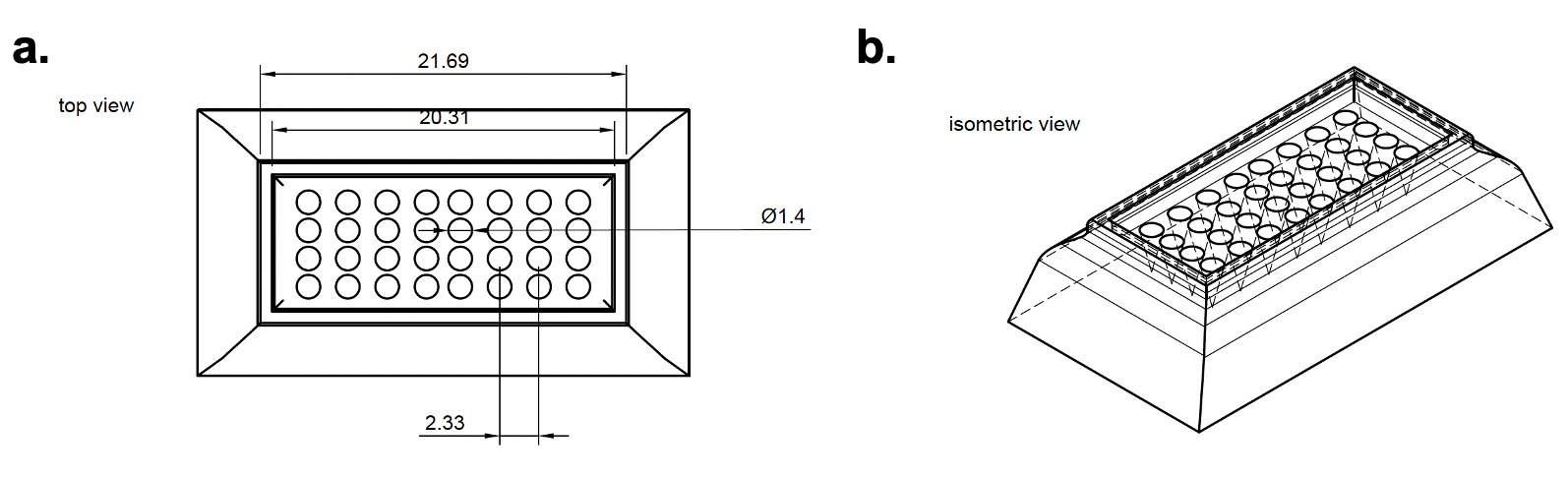


**Figure S2.** Schematic illustrations of PDMS negative mold.

**Table S1.** Summary of microneedle fabrication test conditions.

| Condition ID | Gelatin Conc. | Dehydration Time | Anthocyanin Conc. |
| --- | --- | --- | --- |
| 1 | 5% | 24 h | 0% |
| 2 | 10% | 24 h | 0% |
| 3 | 15% | 8 h | 0% |
| 4 | 15% | 16 h | 0% |
| 5 | 15% | 24 h | 0% |
| 6 | 20% | 24 h | 0% |
| 7 | 15% | 24 h | 0.1% |
| **8** | **15%** | **24 h** | **0.5%** |
| 9 | 15% | 24 h | 0.9% |


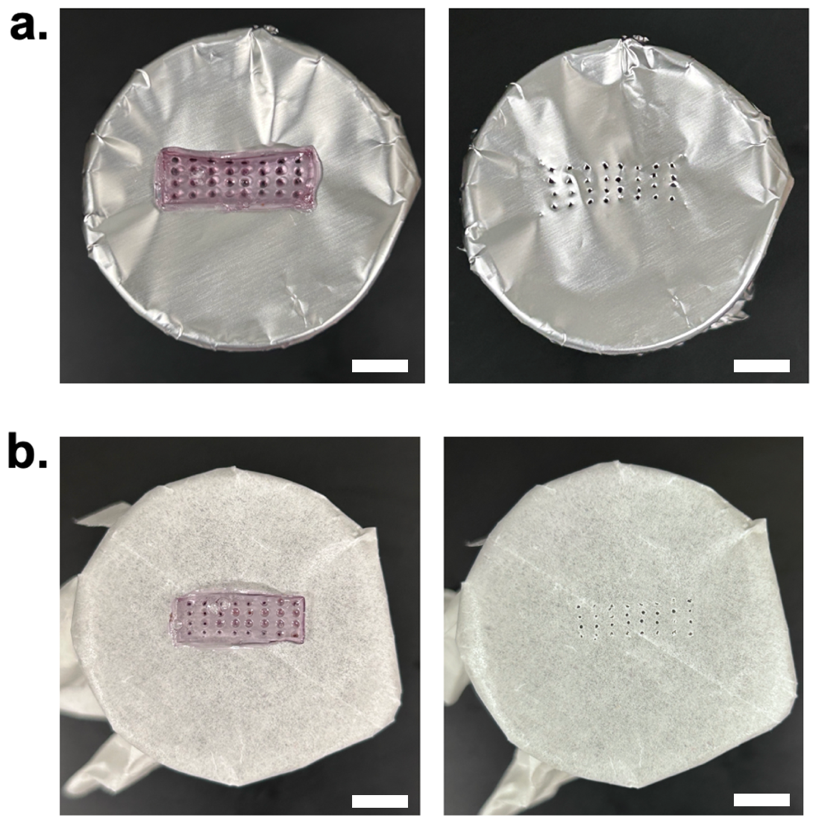


**Figure S3.** Penetration of alternative packaging materials. (a) Application and resultant penetration of tin foil. (a) Application and resultant penetration of wax paper. Scale bar depicts 10 mm.


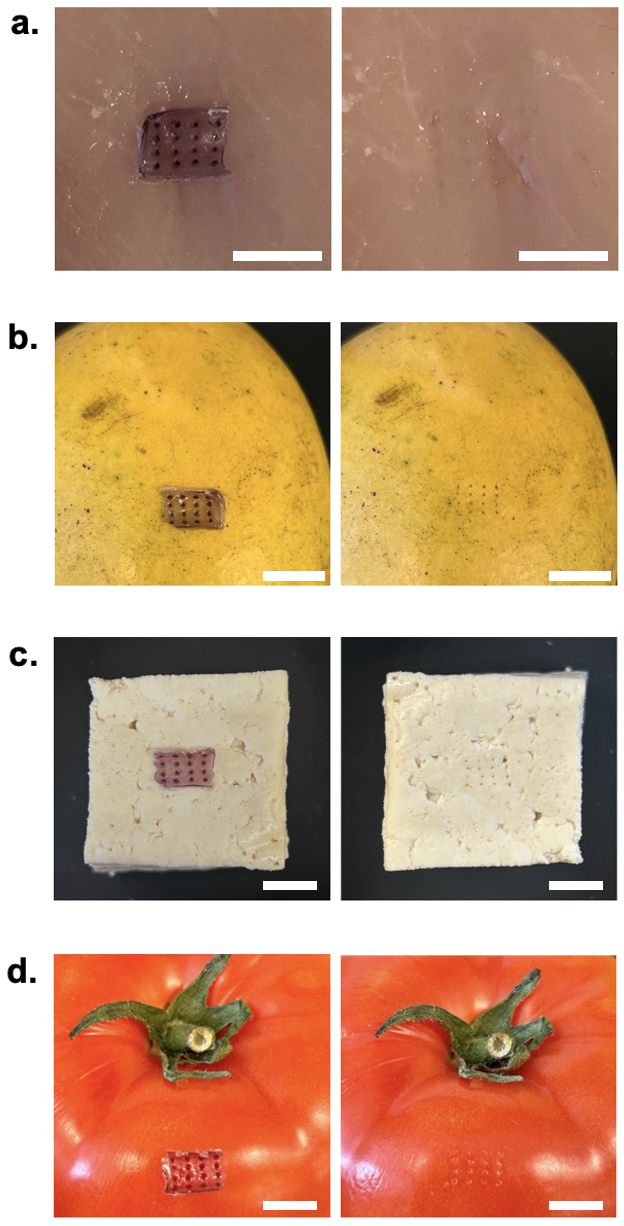


**Figure S4.** Microneedle application and resultant penetration on (a) chicken, (b) mango, (c) tofu, and (d) tomato. Scale bar depicts 10 mm.

**
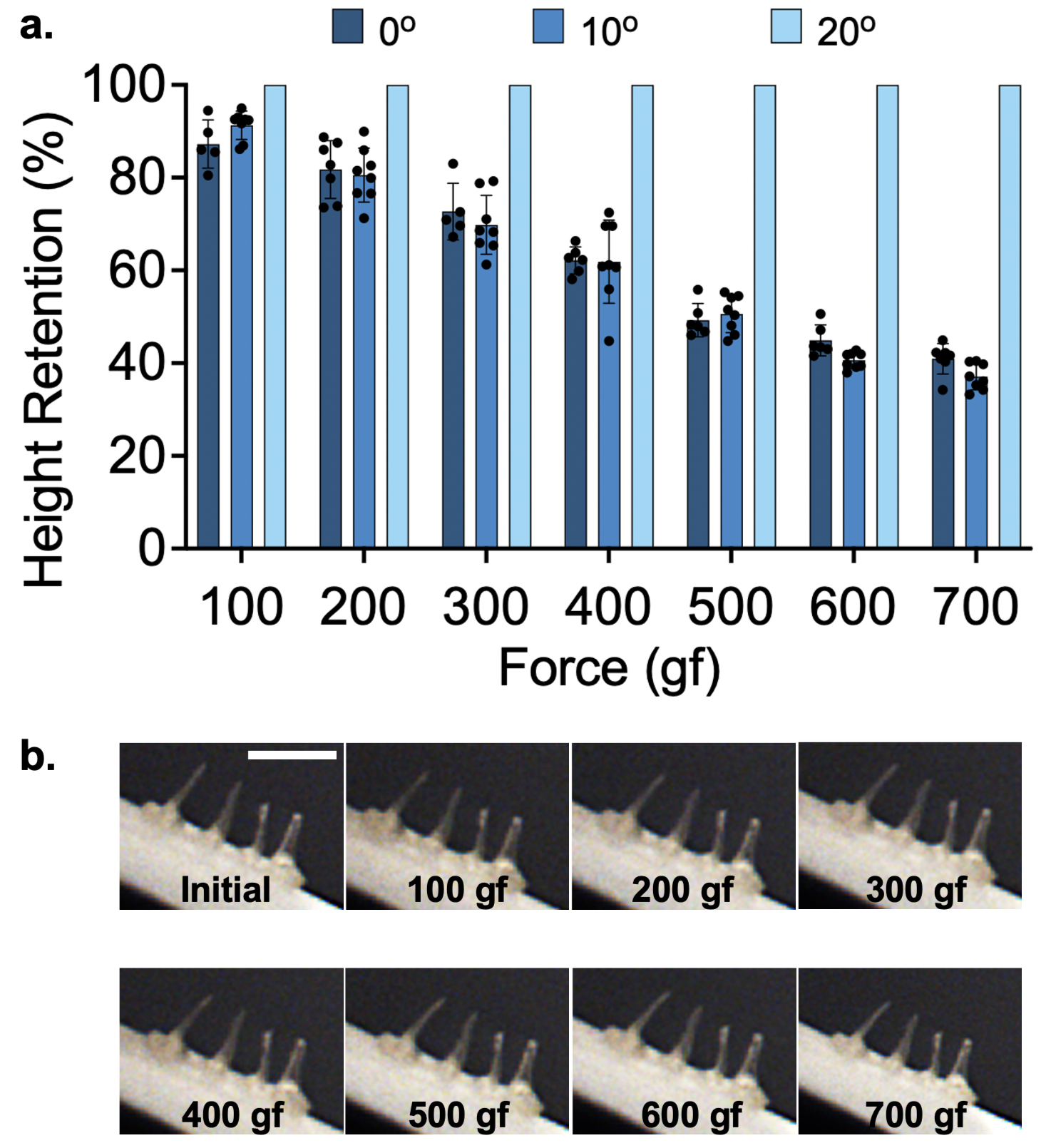
**

**Figure S5.** Mechanical force testing with varied force application angles. (a) Height reduction values across force values ranging from 100 to 700 gf, at angles of 0°, 10°, and 20°. (b) Optical images of retained height when force is applied at 20°. Scale bar depicts 5 mm.

**
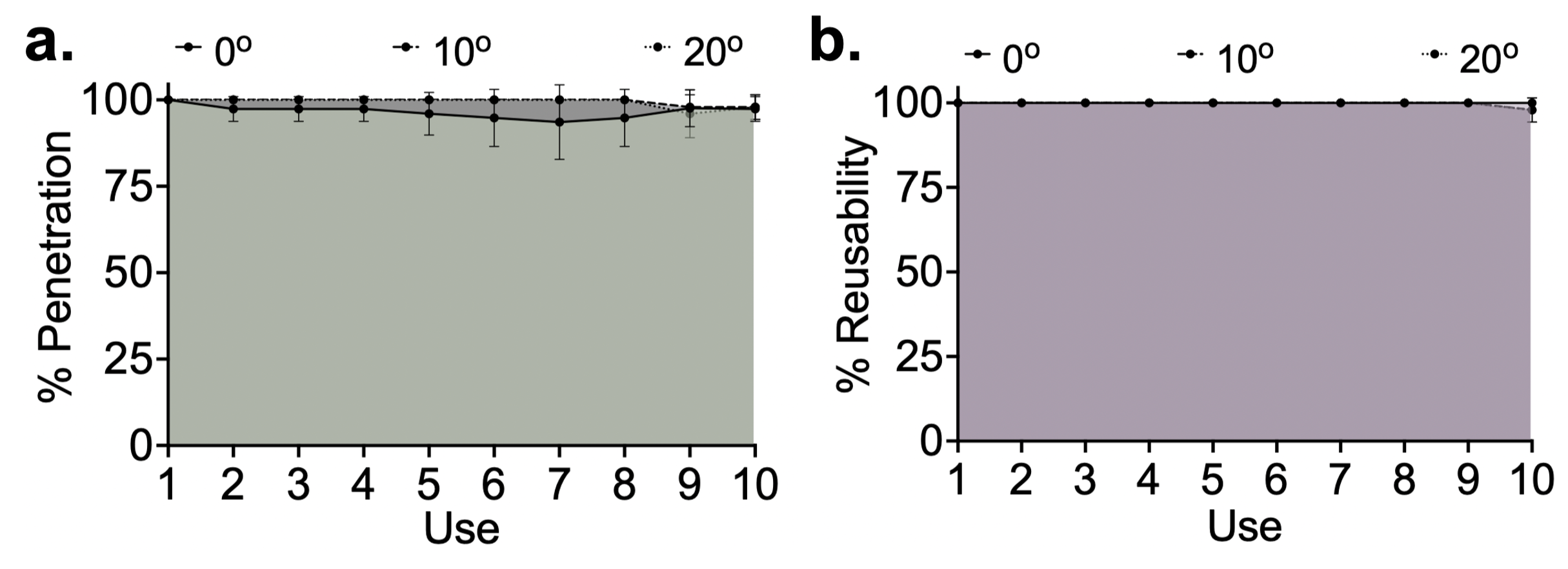
**

**Figure S6.** Varied angles of insertion assessed with regards to (a) penetration rate and (b) reusability.


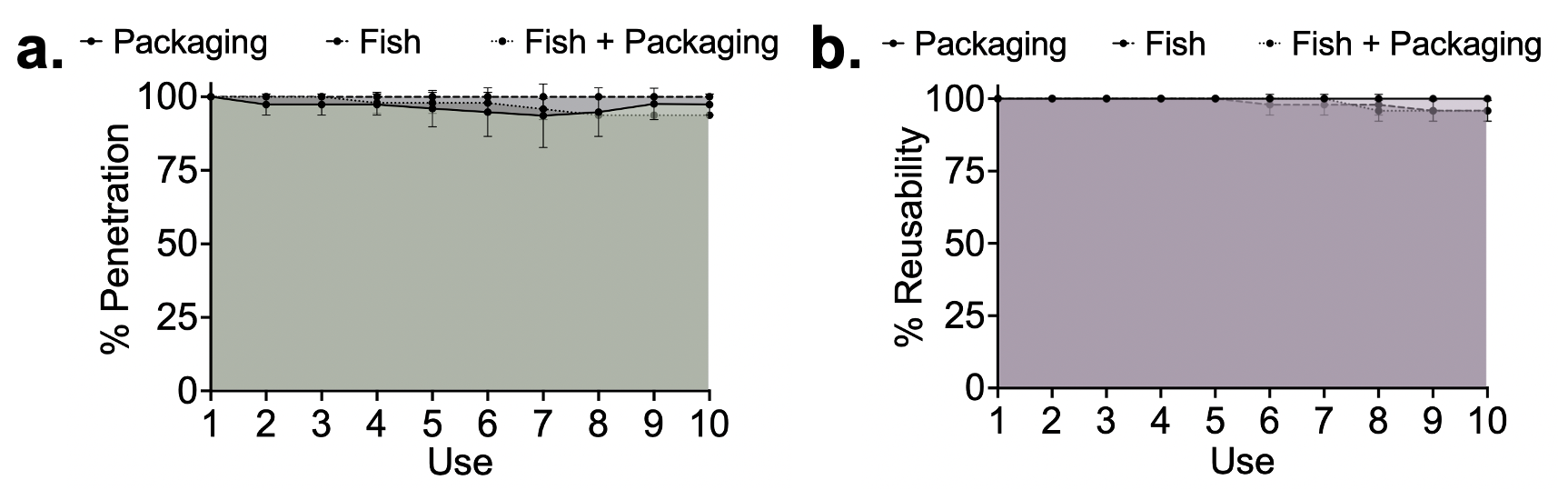


**Figure S7.** Assessment of microneedles against polyethylene packaging film, fish, and polyethylene packaging film + fish with regards to (a) penetration and (b) reusability.


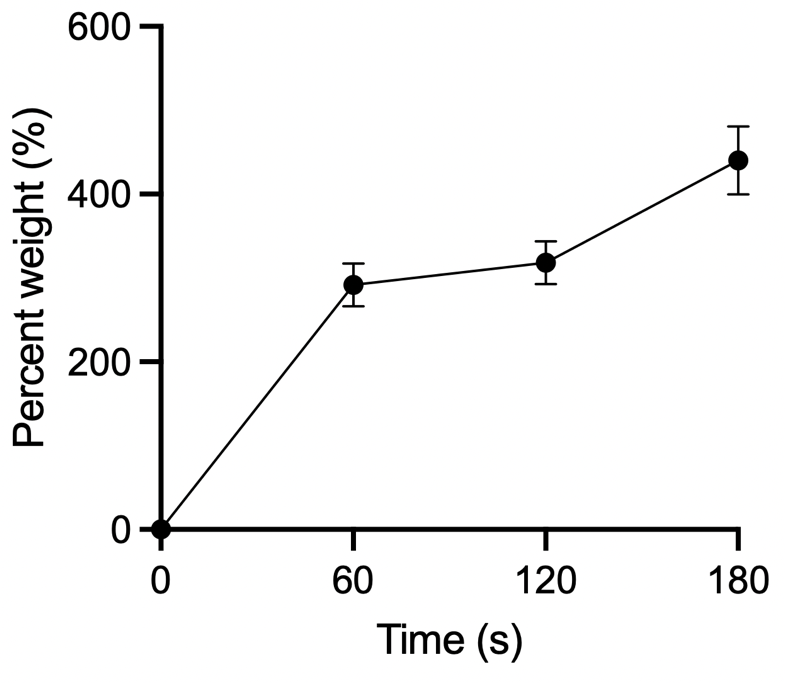


**Figure S8.** Dehydrated gelatin rehydration – defined by weight of water absorbed, across the first three minutes.


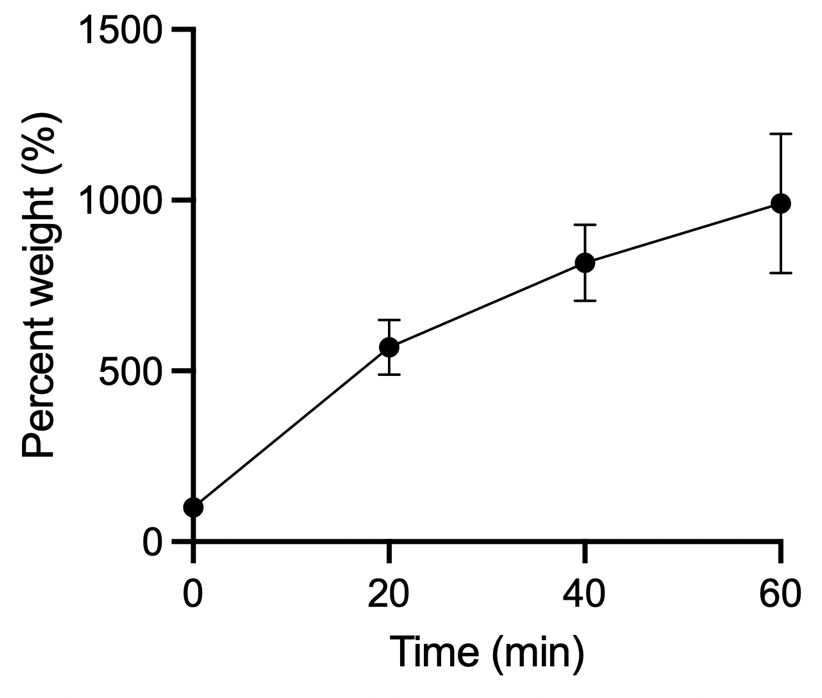


**Figure S9.** Dehydrated gelatin rehydration – defined by weight of water absorbed, across one hour.


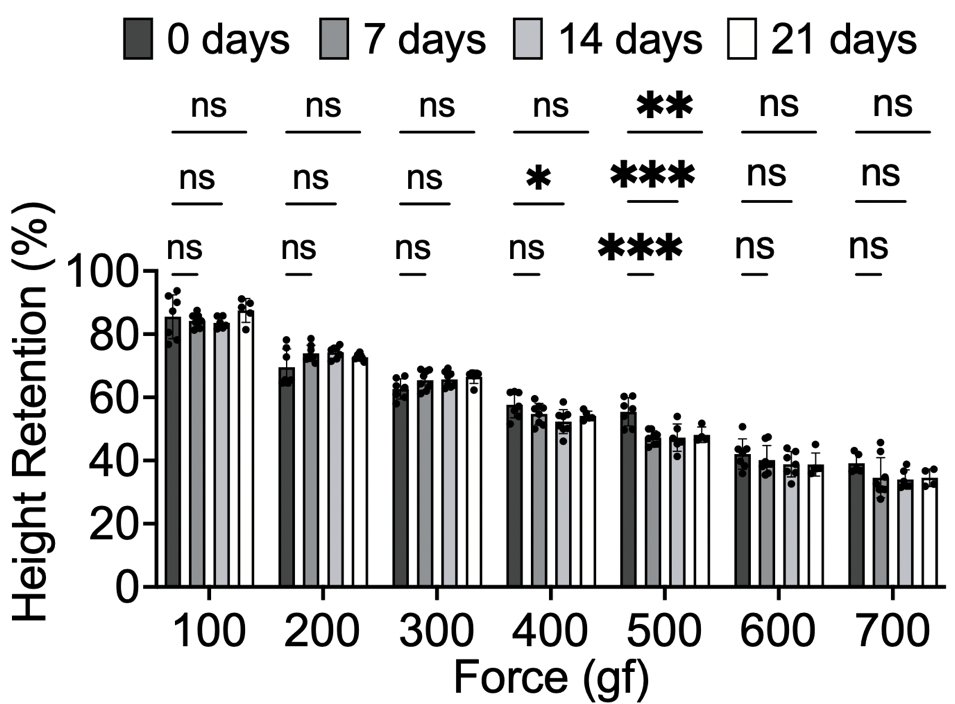


**Figure S10.** Mechanical integrity of 0.9% anthocyanin-embedded microneedles following storage at room temperature for varying lengths of time.


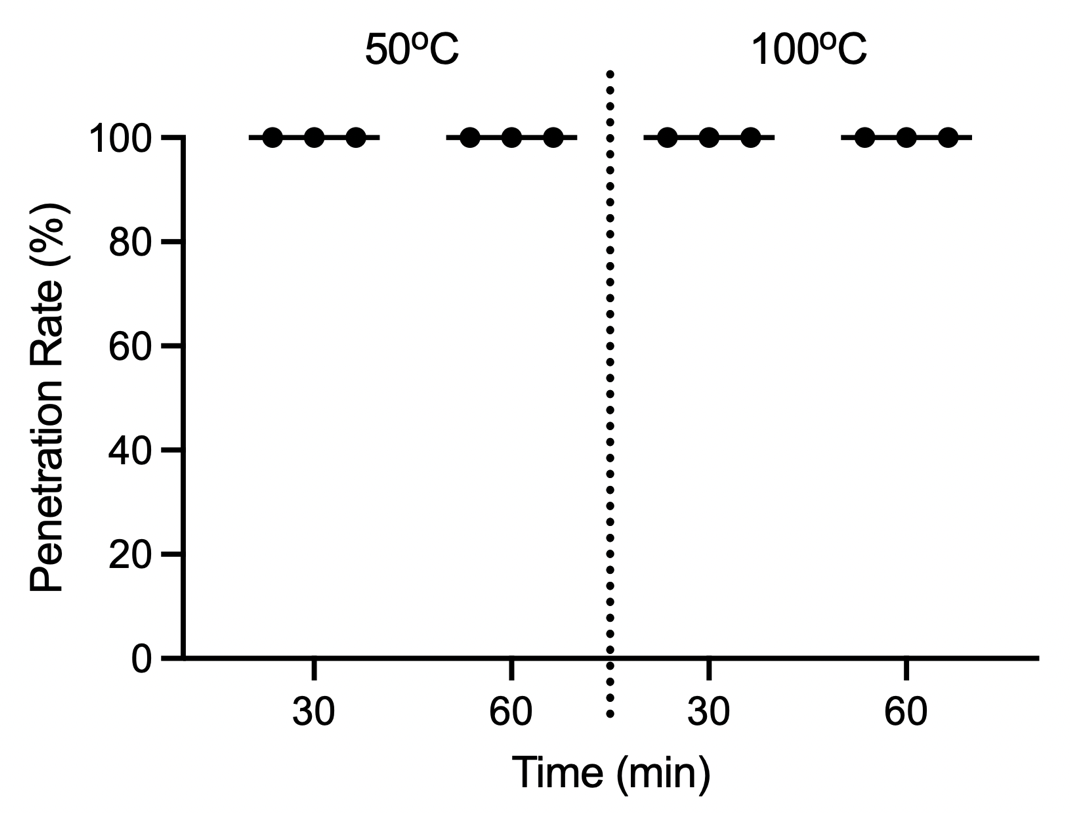


**Figure S11.** Dehydrated gelatin microneedle penetration rates following 30 min and 60 min exposure to high temperature conditions.


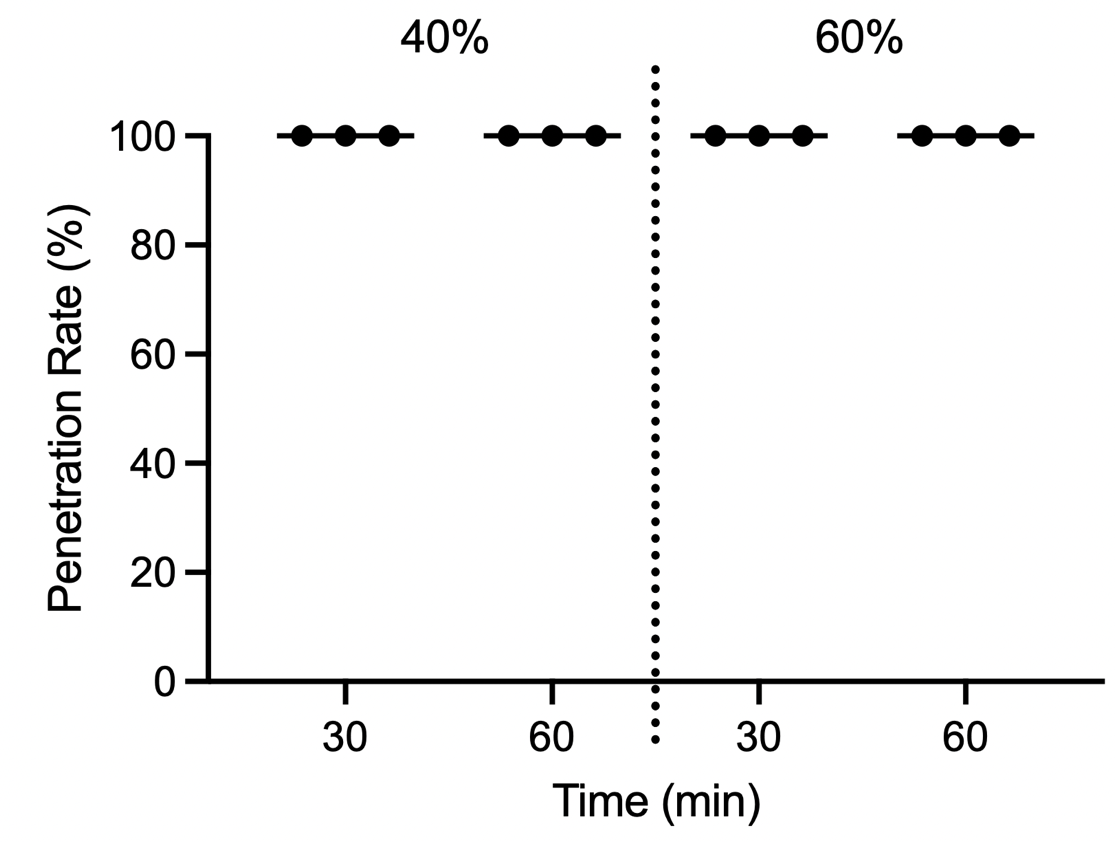
**Figure S12.** Dehydrated gelatin microneedle penetration rates following 30 min and 60 min exposure to varying humidity conditions.


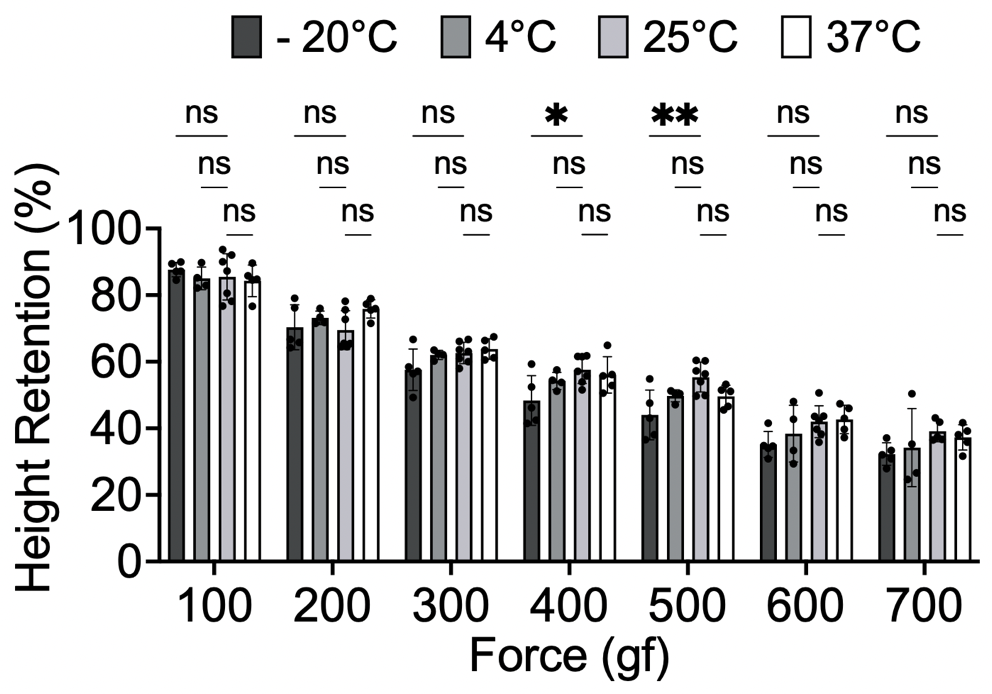


**Figure S13.** Mechanical integrity of 0.9% anthocyanin-embedded microneedles following exposure to diverse temperature conditions.

**Figure S14.** Anthocyanin leaching evaluation following immersion in water.**
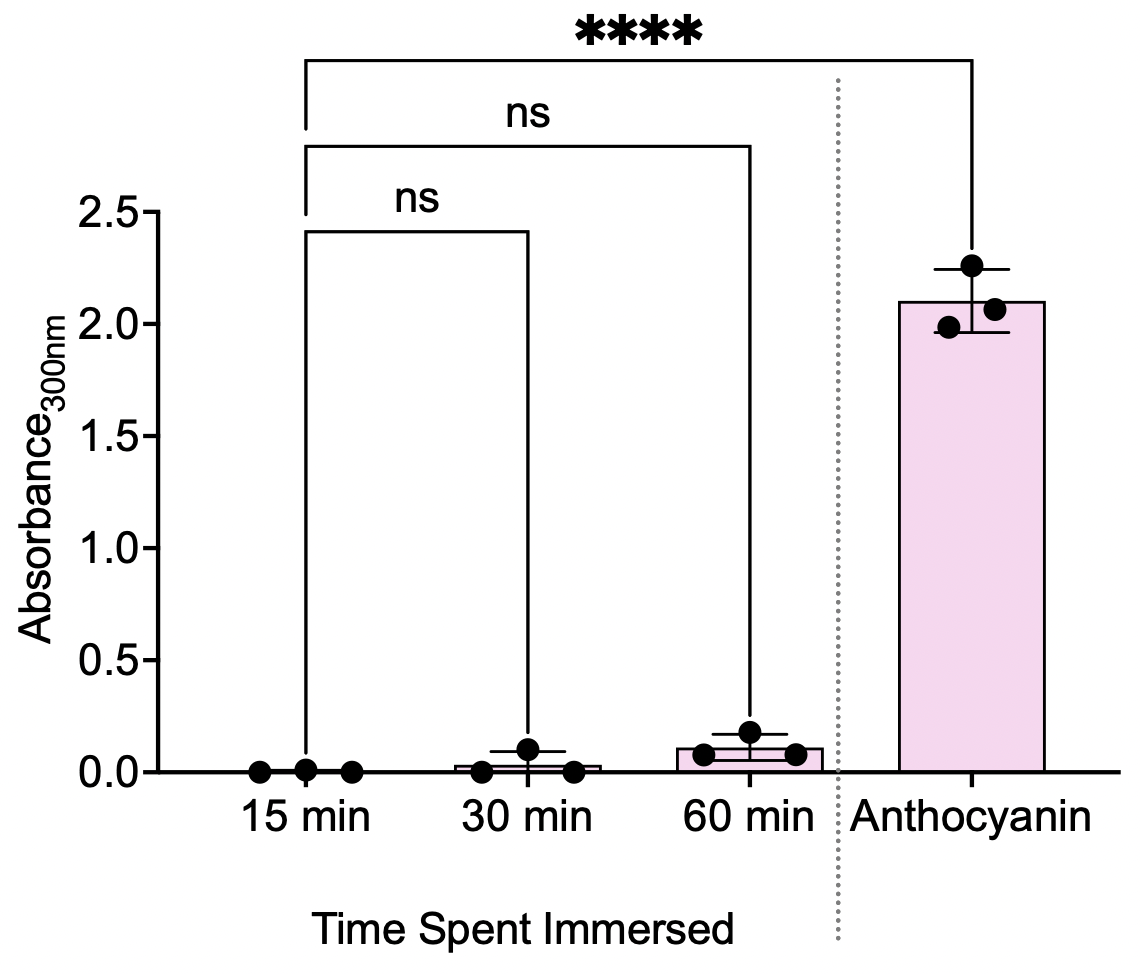
**


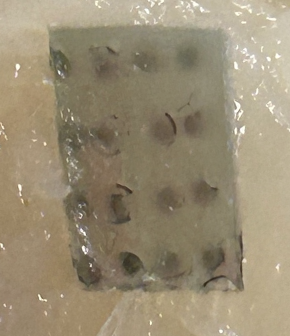

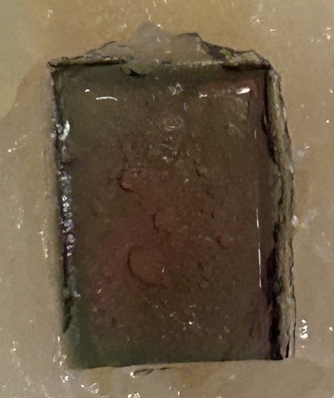


**Figure S15.** Colorimetric response of 0.5% anthocyanin-embedded, dehydrated flat patch (left) versus 0.5% anthocyanin-embedded, dehydrated microneedles after 45 minutes of insertion in an unpackaged, spoiled fish product. Scale bar depicts 5 mm.

**
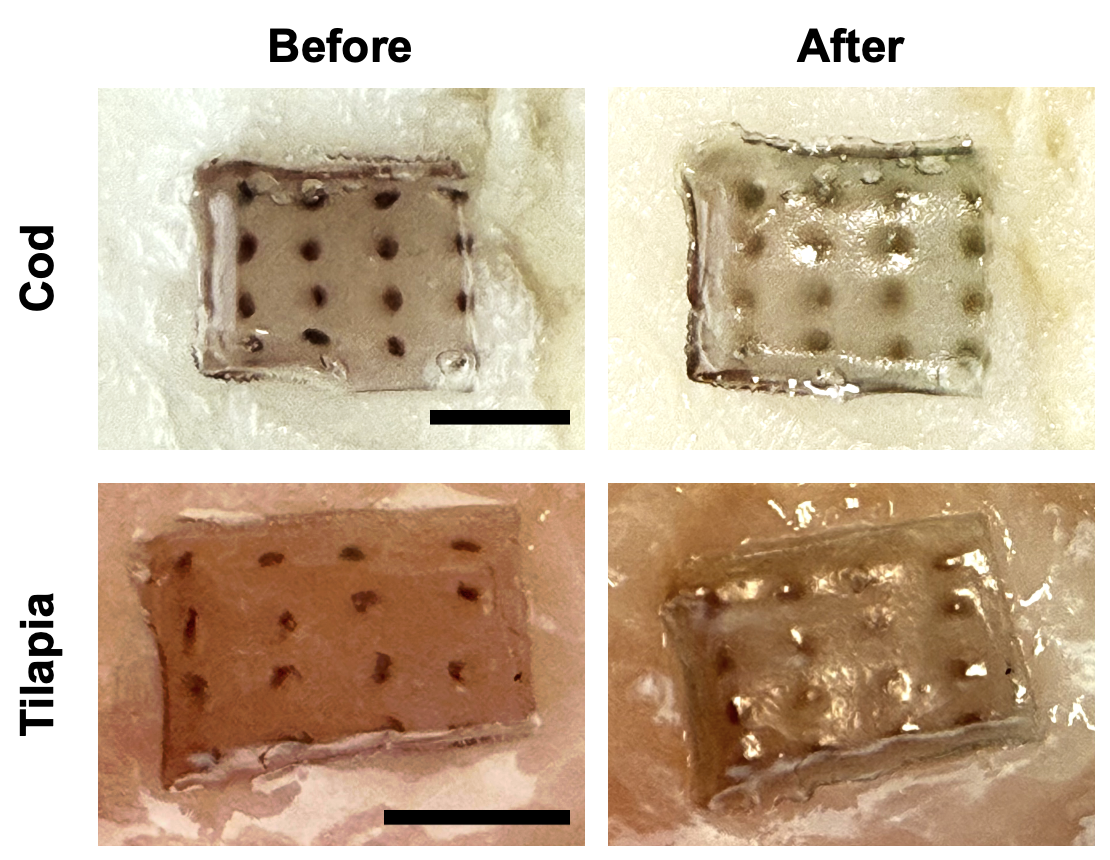
**

**Figure S16.** Spoilage sensing performed on cod and tilapia. Scale bars depict 10 mm.
